# Attitudes of University Students towards Mandatory COVID-19 Vaccination Policies: A Cross-Sectional Survey in Rome, Italy

**DOI:** 10.3390/vaccines11040721

**Published:** 2023-03-23

**Authors:** Antonio Sciurti, Valentina Baccolini, Erika Renzi, Maria Roberta De Blasiis, Leonardo Maria Siena, Claudia Isonne, Giuseppe Migliara, Azzurra Massimi, Corrado De Vito, Carolina Marzuillo, Paolo Villari

**Affiliations:** Department of Public Health and Infectious Diseases, Sapienza University of Rome, 00185 Rome, Italy; antonio.sciurti@uniroma1.it (A.S.); erika.renzi@uniroma1.it (E.R.); mariaroberta.deblasiis@uniroma1.it (M.R.D.B.); leonardo.siena@uniroma1.it (L.M.S.); claudia.isonne@uniroma1.it (C.I.); giuseppe.migliara@uniroma1.it (G.M.); azzurra.massimi@uniroma1.it (A.M.); corrado.devito@uniroma1.it (C.D.V.); carolina.marzuillo@uniroma1.it (C.M.); paolo.villari@uniroma1.it (P.V.)

**Keywords:** vaccines, vaccination, COVID-19, mandatory vaccination

## Abstract

Mandatory vaccination (MV) against COVID-19 is a contentious topic. In this study, we used logistic regression models to identify attitudes among Sapienza University students towards MV for COVID-19. We considered three different scenarios: mandatory COVID-19 vaccination (MCV) for healthcare workers (HCWs) (Model 1), for all people aged ≥ 12 years (Model 2), and for admission to schools and universities (Model 3). We collected 5287 questionnaires over a six-month period and divided these into three groups (September–October 2021, November–December 2021, and January–February 2022). MCV for HCWs was the most strongly supported policy (69.8% in favour), followed by MCV for admission to schools and universities (58.3%), and MCV for the general population (54.6%). In a multivariable analysis, the models showed both similarities and differences. There was no association of socio-demographic characteristics with the outcomes, apart from being enrolled in non-healthcare courses, which negatively affected Models 2 and 3. A greater COVID-19 risk perception was generally associated with a more positive attitude towards MCV, although heterogeneously across models. Vaccination status was a predictor of being in favour of MCV for HCWs, whereas being surveyed in November–February 2022 favoured MCV for admission to schools and universities. Attitudes towards MCV were variable across policies; thus, to avoid unintended consequences, these aspects should be carefully considered by policymakers.

## 1. Introduction

After the introduction of the COVID-19 vaccination into medical practice, addressing vaccine hesitancy (VH) has become one of the main concerns of national and international authorities across the world [1,2]. It has been defined by the World Health Organization’s Strategic Advisory Group of Experts on Immunization as a “delay in acceptance or refusal of vaccination despite availability of vaccination services” [3]. VH is a complex behaviour varying across time and place [4], but already in 2019, prior to the COVID-19 emergency, it was of such concern that it was listed among the top ten threats to global health [5]. The reasons for VH have been studied in recent years [6] and seem to be both multifaceted and culture-specific, but they can be categorized into contextual, individual, group, and vaccine-specific factors [3].

Among the strategies proposed to address the VH phenomenon, one of the most contentious is mandatory vaccination (MV) [7,8]. Historically, MV has mainly been implemented in two groups of people, namely school-aged children and healthcare workers (HCWs). MV for children as a condition for admission to schools was introduced as a response to declining childhood vaccination coverage and the consequent rise of communicable-disease outbreaks both in Europe and the United States [9,10]. With respect to HCWs, some countries mandated seasonal influenza vaccinations because these individuals are recognized as an occupational group at high risk of becoming infected themselves and of infecting patients and colleagues [11,12,13]. These MV policies were mostly successful in increasing vaccination rates [14,15,16], but in both cases have sparked controversy in the population, with legal backlash and opposition from parents and workers [17]. There have also been concerns among experts about the public-health consequences of these policies [18,19,20].

Similarly, despite the increase in COVID-19 vaccination uptake [21] brought about by the MV policies introduced for these vaccines [22,23], mandatory COVID-19 vaccination (MCV) has generated concern and opposition in the general population [23] and extensive debate among experts [24]. Within this context, even though COVID-19 vaccination as a requirement for access to educational institutions is uncommon [25,26,27], university students were deeply impacted by the restrictions imposed by the pandemic, leading them to have different opinions on MCV [28]. To date, a few studies have investigated the attitudes of university students towards MV, but they have mostly focused on healthcare students or trainees [29,30]. In addition, to the best of our knowledge, no study has assessed attitudes towards different MCV policies. Therefore, the aim of this study was to investigate attitudes towards MCV policies in a large sample of Italian university students enrolled at Sapienza University of Rome, distinguishing between three scenarios (i.e., MCV for HCWs, for all people aged 12 or above, and for people attending schools or universities) and identifying their predictors. 

## 2. Materials and Methods

### 2.1. Setting and Participants

This cross-sectional study was carried out on students who participated in the free SARS-CoV-2 screening campaign offered by Sapienza University, a public university located in Rome, Italy, and one of the largest in Europe by number of enrolments [31]. The testing program has been described elsewhere [31]. Briefly, the student swab service ran on Monday–Thursday from 8:30 AM to 4:00 PM from 8 September 2021 to 22 December 2021, with a break over Christmas. It restarted on 10 January 2022 and ran Monday–Thursday from 8.30 AM to 11:30 AM until the end of February 2022.

While students were waiting for their turn at the screening site, they were invited to voluntarily take part in this online survey accessible via smartphone through a QR code. Whereas the screening service could be used multiple times, it was only possible to fill out the questionnaire once. The study was performed in accordance with the World Medical Association Declaration of Helsinki. Participants were asked for their consent and were guaranteed anonymity in the information collected. The institutional ethics board of the Umberto I teaching hospital/Sapienza University of Rome approved this study (protocol N. 911/2021).

### 2.2. Questionnaire

The questionnaire was derived from other studies [29,30]. It consisted of 19 questions grouped into four sections and took approximately five minutes to fill out. 

The first section of the questionnaire collected sociodemographic information: age, gender, nationality, faculty, year of study, financial situation (i.e., how well do you get to the end of the month with the financial resources at your disposal?). Students were also asked whether they had already had COVID-19 and, if the answer was yes, how severe their symptoms had been (i.e., asymptomatic, mild symptoms, moderate symptoms, severe symptoms).

The second section focused on the experience of COVID-19 vaccination. We asked whether students had received at least one dose of a COVID-19 vaccine. Students answering in the negative were asked the main reasons for not being vaccinated. One question investigated the occurrence of adverse events in vaccinated students (i.e., no adverse events, mild adverse events, moderate or severe adverse events). 

In the third section, students were asked to rate from 0 (not at all) to 10 (extreme) the (perceived) severity of the COVID-19 disease, their concern about the COVID-19 emergency, and their fear of infecting people in the community and of becoming infected. One question also investigated to what extent students viewed the vaccines as an effective way to end the pandemic (on a scale from 0 [not at all] to 10 [extremely]).

The last section explored students’ attitudes towards three different MCV policies: (i) MCV for HCWs, (ii) MCV for all people aged 12 and above without medical contraindications, and (iii) MCV for access to schools and universities. Specifically, students were asked to rate how strongly they were in favour of mandating vaccination on a scale from 0 (not at all in favour) to 10 (extremely in favour) in each of the abovementioned contexts.

### 2.3. Statistical Analysis

Descriptive statistics were obtained using median and interquartile range, or mean and standard deviation, for continuous variables and proportions for dichotomous and categorical variables. Students were considered either Italian or non-Italian. Faculties were categorised into three areas: healthcare (e.g., medicine, nursing), science and technology (e.g., mathematics, biology), or social sciences and humanities (e.g., law, economics). Students were divided into two groups according to their year of study: first- and second-year students (i.e., who started their university career during the pandemic) vs. third-year students or above. Questionnaires were divided into three groups according to the date of survey completion: (i) from 8 September to 28 October 2021; (ii) from 2 November to 22 December 2021; and (iii) from 10 January to 24 February 2022 (i.e., after the Christmas break). Given the high prevalence of students that were completely in favour of mandating COVID-19 vaccination (i.e., that answered 10/10 to the question “on a scale from 0 [not at all] to 10 [extremely], how strongly are you in favour of mandating COVID-19 vaccination…”), the answers to the three policies (i.e., MCV for HCWs, for all people aged 12 and above without medical contraindications, and for school and university admission) were dichotomized into two cohorts: being completely in favour of mandating vaccination against COVID-19 (hereafter referred to as “positive attitude”) vs. being partially or not being in favour of mandating vaccination against COVID-19 (hereafter referred to as “negative attitude”).

In the univariate analysis, for each outcome, the Mann–Whitney U test was used to compare continuous variables for the two cohorts of MCV attitudes, whereas Pearson’s chi-squared or Fisher’s exact test was used for dichotomous and categorical variables. Then, a multivariable logistic regression model was built to identify predictors of a positive attitude towards each MCV policy considered: i.e., (i) MCV for HCWs, (ii) MCV for all people aged 12 and above without medical contraindications, and (iii) MCV for school and university admission. Variables were included in the model based on expert opinion [32]. Multicollinearity was checked using a variance inflation factor of 5 as the threshold [33]. The Hosmer and Lemeshow test was used to evaluate the goodness of fit of the model [34]. As a result, the final models were composed of the following variables: survey period (categorical), vaccination status (dichotomous), age (continuous), gender (dichotomous), nationality (dichotomous), area of study (categorical), year of study (dichotomous), finances (dichotomous, i.e., having some or many financial difficulties vs. managing well enough or very well), previous COVID-19 infection (categorical), perceived COVID-19 severity (continuous), concern about the COVID-19 emergency (continuous), fear of becoming infected (continuous), fear of infecting people in the community (continuous), and viewing the vaccine as an effective way to end the pandemic (continuous). Adjusted odds ratios (aORs) and 95% confidence intervals (CIs) were calculated. All analyses were performed using Stata (StataCorp LLC, 4905 Lakeway Drive, College Station, TX 322, USA), version 17.0. A two-sided *p*-value < 0.05 was considered statistically significant.

## 3. Results

During the screening campaign, 7329 students were tested for SARS-CoV-2 at least once; of these, 5287 answered the questionnaire. This breaks down to 1240 students answering in the first survey period (daily mean: 41.3, range 17–102), 2961 students in the second survey period (daily mean: 105.8, range 46–161), and 1086 students in the third survey period (daily mean: 38.8, range 20–96). The overall response rate was 72.1%, with the highest proportion in November–December 2021 (77.4%), followed by January–February 2022 (76.2%) and September–October 2021 (59.6%).

The average age of participating students was 23.9 ± 4.5 years, and 67.7% were females (Table 1). Most students were Italian (87.6%). Almost 60% of the students were attending the first or second year of their course, and the largest category of students were those enrolled in healthcare-related faculties (37.7%). In terms of finances, approximately half of the participants reported getting to the end of the month well enough, and roughly 20% reported managing very well, while the remaining 30% had some or many financial difficulties. Regarding past COVID-19 infection, almost 90% of the participants reported not having been infected, but this value fluctuated across the three time periods, with the highest proportion in November–December 2021 (91.3%) and the lowest in January–February 2022 (82.3%).

As for COVID-19 vaccination status, most students reported being vaccinated against SARS-CoV-2 with at least one dose of the vaccine (98.1%), with the proportion of unvaccinated students decreasing over time (Table 2). Among the reasons provided for not being vaccinated, 32.0% reported a lack of belief in the safety or effectiveness of vaccines against COVID-19, and 14.6% did not consider themselves at risk or preferred to obtain natural immunity through infection, while the remainder stated they had already had COVID-19 (20.4%), were waiting to get vaccinated (11.7%), or were suffering from a clinical condition with contraindications to COVID-19 vaccination or were waiting for further medical assessment (16.5%). Approximately one in ten respondents reported experiencing moderate or severe adverse events associated with vaccination. Regarding perceptions and experiences of the COVID-19 pandemic, slightly higher values of perceived COVID-19 severity and concern about the emergency were observed in November–December 2021 (7.8 and 7.7, respectively) compared to the other two periods of the campaign (September–October: 7.6 and 7.2; January–February: 7.1 and 7.3, respectively). Similarly, the highest average values of fear of becoming infected and infecting people in the community were registered in November–December 2021 (7.6 and 8.2), as well as viewing vaccines as an effective way to end the pandemic (9.0). 

As for the attitudes towards MCV policies, the highest proportion of students were in favour of MCV for HCWs (69.8%), followed by MCV for admission to schools and universities (58.3%), and MCV for all people aged 12 and above (54.6%) (Table 2). 

**Figure 1:**
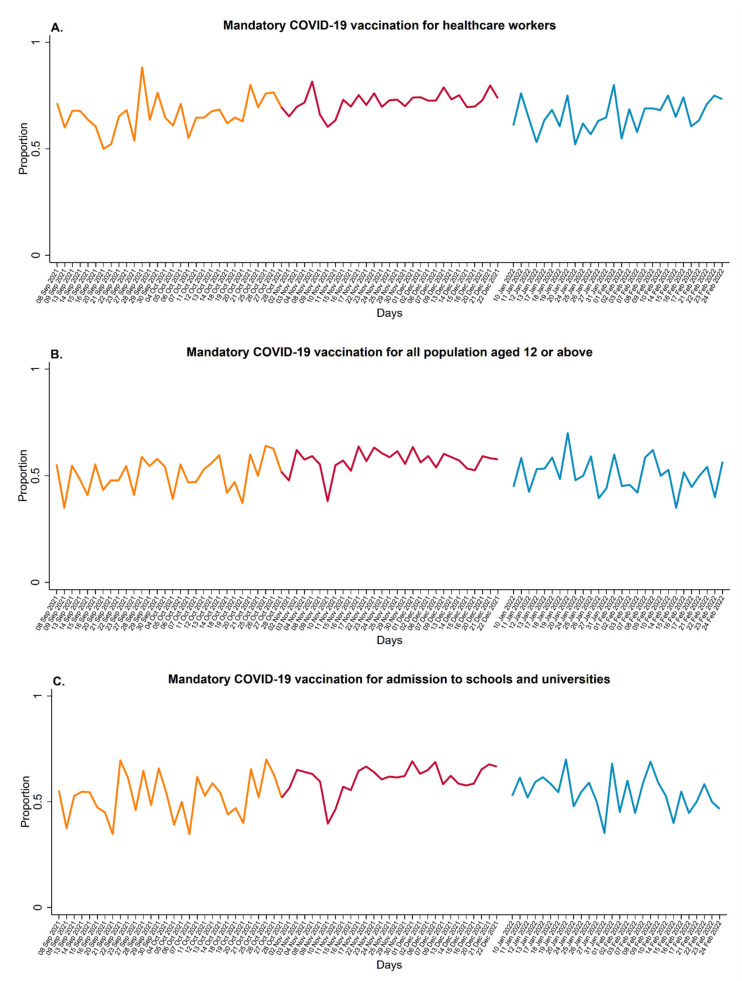
Proportion of students with a positive attitude towards mandatory COVID-19 vaccination policies by survey period (i.e., from 8 September to 31 October 2021: orange line; from 2 November to 22 December 2021: red line; and from 10 January to 24 February 2022: blue line).

The daily proportion of students being completely in favour of MCV over the SARS-CoV-2 screening campaign is illustrated in Figure 1. As for the attitude towards MCV for HCWs (Figure 1A), the proportion of students in favour slightly increased from September–October 2021 to November–December 2021 and clearly decreased in the last survey period, whereas the attitude towards MCV for all people aged 12 and above and MCV for admission to schools and universities showed similar but more fluctuating trends (Figure 1B,C, respectively).

A univariate analysis by attitude towards the three MCV policies showed significant differences in most of the variables considered (Supplementary Tables S1 and S2). In the multivariable analysis, the models showed some similarities in terms of significant predictors (Table 3). Specifically, age, gender, nationality, year of study, finances, and previous COVID-19 infection status did not seem to be associated with any of the three outcomes, whereas a higher value of perceived COVID-19 severity, being afraid of infecting people in the community, and viewing the vaccine as an effective way to end the pandemic increased the odds of a positive attitude towards all MCV policies. By contrast, compared to students enrolled in healthcare-related faculties, students enrolled in Science & Technology faculties had lower odds of being completely in favour of MCV for admission to schools and universities only (Model 3, aOR: 0.85, 95% CI: 0.73–0.99); students enrolled in Social Sciences & Humanities faculties were more doubtful about MCV both for admission to schools and universities (Model 3, aOR: 0.80, 95% CI: 0.69–0.93) and for people aged 12 and above (Model 2, aOR: 0.81, 95% CI: 0.69–0.94), whereas no difference was found in the area of study for MCV for HCWs (Model 1). As for the other differences, being vaccinated had a positive association with MCV in Model 1 only (aOR: 2.47, 95% CI: 1.36–4.48), being more afraid of becoming infected was significantly associated with Model 3 only (aOR: 1.06, 95% CI: 1.03–1.10), while having greater concern about the emergency increased the odds of the outcome in both Models 2 and 3 (aOR: 1.08, 95% CI: 1.04–1.13; and aOR: 1.09, 95% CI: 1.04–1.13, respectively). Lastly, time of questionnaire completion did not show significant associations in any model except for Model 3, in which being surveyed between November–December 2021 and January–February 2022 resulted in greater odds of a positive attitude towards MCV for admission to schools and universities (aOR: 1.28, 95% CI: 1.10–1.49, and aOR: 1.21, 95% CI: 1.01–1.46, respectively).

## 4. Discussion

After extensive consultation with experts and the general public, Italy, like other countries, made COVID-19 vaccination mandatory for specific categories of workers—such as healthcare workers, school and university personnel, the military, law enforcement, and public servants [35,36]. Workers that did not comply with MCV were suspended without pay until they got vaccinated [37]. MCV was then extended to all adults aged 50 years or over, with a 100-Euro fine for those not complying [37]. In this survey, at least half of the students had a positive attitude toward the MCV scenarios proposed, with the most supported policy being MCV among HCWs. Moreover, such a policy showed less variability in its associated predictors in the multivariable analysis, suggesting that MCV for HCWs was more widely accepted than the other two mandates. These findings support the idea that HCWs are deemed responsible for patient safety, not only by healthcare professionals themselves, as observed in studies on MV for seasonal influenza [38,39], but also by a broader strata of society, including our participants. This result is also in line with those studies that assessed attitudes towards MCV among healthcare students and professionals, where support for MCV for HCWs was found to be reasonably high [40,41,42,43,44,45]. In addition, the positive association with vaccination status found only for this MCV policy might strengthen the idea that our vaccinated students, the vast majority of the sample, were driven by a strong sense of responsibility towards society that led them to consider the act of vaccinating HCWs as essential for the protection of others [46].

Notably, we found that MCV for access to education was slightly more accepted than MCV for all people aged 12 or older, probably the result of a sense of vulnerability that made students advocate for a self-protection policy [28]. At the time of the survey, despite Italian university students not being included in the vaccination mandates, they were required to show a European Digital Green Certificate (“Green Pass”) to attend in-person activities on campus [47,48]. However, this university policy may not have been sufficient to make students feel entirely safe, especially during some phases of the pandemic. Indeed, in line with the literature [49], which shows attitudes towards MCV policies changing over time, in our study, the students’ attitudes towards MCV for access to educational institutions were particularly variable, being significantly higher during November 2021–February 2022; this was a time when there were peaks in virus circulation due to the spread of the Delta variant and the emergence of Omicron across the country [50]. Arguably, the ubiquity of SARS-CoV-2 may have led to an increased perception of the risks associated with COVID-19, which may also explain why the fear of becoming infected was positively associated with support for COVID-19 MV for access to schools and universities, but not for the other mandates, i.e., it motivated people to become more in favour of the introduction of measures, including MV, to counteract the pandemic in their own specific context [51,52]. 

However, a few similarities were found across the three policies. For instance, higher risk perception was a predictor of positive attitudes towards all MCV policies, probably because it made students more willing to accept measures, including MCV in general, which might prevent infection of themselves and others. In addition, given that another common predictor was fear of infecting other people, our results confirm that being in favour of MCV is affected by considerations of both personal and societal health benefits [53]. Unsurprisingly, we also found that vaccine confidence was consistently related to a positive attitude towards the three outcomes, underlining the importance of limiting disinformation on COVID-19 vaccines to increase their public acceptance [6]. This may have a twofold effect: firstly, it might reduce VH, which should be a central aim of any communication campaign [54,55]; and secondly, it might promote acceptance of MV, a policy that should be introduced only after careful consideration [56]. 

Despite the literature reporting that, for both HCWs [57] and the general population [49,58], the female gender is frequently associated with a less supportive attitude towards MCV, we did not detect any sex difference in attitudes. Neither did we find an association with nationality, financial situation, or previous COVID-19 infection, probably reflecting a mitigation in attitudes in our sample as the pandemic progressed. A lack of association of attitudes with increasing age was probably because the age range in our sample was relatively narrow compared to other studies [49,57]. Lastly, with regard to education, it was not the year of study but rather the area of enrolment that revealed some discrepancies across MCV policies. In general, non-healthcare students seemed to be more doubtful about MCV for either the general population, or for admission to schools and universities, or both, especially in the case of students attending Social Sciences & Humanities faculties. A possible explanation for this finding is that non-healthcare students have a lower level of health literacy than healthcare students, which may increase their VH and decrease their acceptance of MCV [6,59,60], but further research is needed to confirm this hypothesis.

This study has some strengths and limitations. Firstly, the cross-sectional design hindered the opportunity to draw causal links between attitudes towards MCV policies and the associated factors. Secondly, despite the good response rate we achieved, participants were recruited from those attending in-person activities and participating in the SARS-CoV-2 screening campaign; therefore, our sample may be not entirely representative of Sapienza University students. However, the fact that the survey was conducted when the vaccination campaign was at its height for young people, at a time when the university was strongly encouraging students to attend the campus, may have limited this effect. Nevertheless, to the best of our knowledge, this is the first study to investigate attitudes towards MCV in a broad sample of Italian university students, distinguishing three different MCV policies and comparing predictors across them. In addition, given the considerable time span of the study, we were able to track student attitudes over time, thereby investigating any potential change in relation to the pandemic trajectory. 

## 5. Conclusions

Mandating COVID-19 vaccination has been a subject of considerable debate in Italy. In our study, attitudes of university students towards MCV depended on the population considered, confirming the notion that the MV concept is context-specific and multifaceted. Indeed, while a few predictors were similar across the three proposed policies, such as risk perception and confidence in vaccines, others were not, including the area of study, vaccination status, and time of survey completion. Since MV could have unintended consequences, these aspects need to be carefully considered by policymakers. 

## Figures and Tables

**Table 1:** Students’ sociodemographic characteristics, overall and by survey period. Results are expressed as mean (standard deviation, SD), median (interquartile range, IQR), or frequency (percentage, %). COVID-19: coronavirus disease 2019.

	Total	September– October 2021	November– December 2021	January– February 2022
	N = 5287	N = 1240	N = 2961	N = 1086
Age, years				
Mean (SD)	23.9 (4.5)	24.5 (5.2)	23.4 (4.0)	24.6 (4.5)
Median (IQR)	23.1 (21.2–25.2)	23.2 (21.5–25.8)	22.7 (21.0–24.6)	23.8 (21.8–26.1)
Gender, N (%)				
Female	3580 (67.7)	823 (66.4)	2015 (68.1)	742 (68.3)
Male	1707 (32.3)	417 (33.6)	946 (31.9)	344 (31.7)
Nationality, N (%)				
Italian	4632 (87.6)	1036 (83.5)	2651 (89.5)	945 (87.0)
Non-Italian	655 (12.4)	204 (16.5)	310 (10.5)	141 (13.0)
Area of study, N (%)				
Healthcare	1994 (37.7)	463 (37.3)	1152 (38.9)	379 (34.9)
Science & Technology	1628 (30.8)	315 (25.4)	960 (32.4)	353 (32.5)
Social Sciences & Humanities	1665 (31.5)	462 (37.3)	849 (28.7)	354 (32.6)
Year of study, N (%)				
First or second	3288 (62.2)	719 (58.0)	1833 (61.9)	736 (67.8)
Third or above	1999 (37.8)	521 (42.0)	1128 (38.1)	350 (32.2)
Finances, N (%)				
Many difficulties	237 (4.5)	69 (5.6)	126 (4.3)	42 (3.9)
Some difficulties	1339 (25.3)	343 (27.7)	706 (23.8)	290 (26.7)
Managing well enough	2753 (52.1)	615 (49.6)	1565 (52.9)	573 (52.8)
Managing very well	958 (18.1)	213 (17.2)	564 (19.0)	181 (16.7)
Previous COVID-19 infection, N (%)				
No infection	4709 (89.1)	1113 (89.8)	2702 (91.3)	894 (82.3)
Asymptomatic or mild symptoms	501 (9.5)	112 (9.0)	227 (7.7)	162 (14.9)
Moderate or severe symptoms	77 (1.5)	15 (1.2)	32 (1.1)	30 (2.8)

**Table 2:** Students’ COVID-19 vaccination experience, COVID-19 risk perception, and attitude towards mandatory COVID-19 vaccination, overall and by survey period. Results are expressed as mean (standard deviation, SD) or frequency (percentage, %). COVID-19: coronavirus disease 2019.

	Total	September– October 2021	November– December 2021	January– February 2022
	N = 5287	N = 1240	N = 2961	N = 1086
Vaccination status, N (%)				
Unvaccinated	103 (1.9)	54 (4.4)	43 (1.5)	6 (0.6)
Vaccinated	5184 (98.1)	1186 (95.6)	2918 (98.5)	1080 (99.4)
Reasons for not getting vaccinated (N = 103), N (%)				
I am suffering from a clinical condition with contraindications to COVID-19 vaccination/waiting for further medical assessment to get vaccinated	17 (16.5)	9 (16.7)	7 (16.3)	1 (16.7)
I’ve already had COVID-19	21 (20.4)	7 (13.0)	12 (27.9)	2 (33.3)
I’ve booked the vaccination/I am waiting to get vaccinated	12 (11.7)	7 (13.0)	4 (9.3)	1 (16.7)
I don’t consider myself at risk/prefer to obtain natural immunity to COVID-19	15 (14.6)	10 (18.5)	4 (9.3)	1 (16.7)
I don’t believe in the safety/effectiveness of vaccines against COVID-19	33 (32.0)	21 (38.9)	12 (27.9)	0 (0.0)
No reason given	5 (4.9)	0 (0.0)	4 (9.3)	1 (16.7)
Vaccine-adverse event (N = 5184), N (%)				
No adverse event	1792 (34.6)	415 (35.0)	1018 (34.9)	359 (33.2)
Mild adverse event	2842 (54.8)	638 (53.8)	1589 (54.5)	615 (56.9)
Moderate or severe adverse event	550 (10.6)	133 (11.2)	311 (10.7)	106 (9.8)
Perceived COVID-19 severity, mean (SD)	7.6 (1.9)	7.6 (2.0)	7.8 (1.8)	7.1 (2.0)
Concern about the COVID-19 emergency, mean (SD)	7.5 (2.0)	7.2 (2.2)	7.7 (1.9)	7.3 (2.1)
Being afraid of infecting people in the community, mean (SD)	8.0 (2.3)	7.8 (2.5)	8.2 (2.2)	7.7 (2.4)
Being afraid of becoming infected, mean (SD)	7.4 (2.5)	7.3 (2.7)	7.6 (2.5)	7.2 (2.6)
Viewing the vaccine as an effective way to end the pandemic, mean (SD)	8.8 (2.0)	8.6 (2.2)	9.0 (1.8)	8.6 (2.1)
Attitude towards mandatory COVID-19 vaccination for healthcare workers, N (%)				
Negative attitude	1597 (30.2)	416 (33.5)	805 (27.2)	376 (34.6)
Positive attitude	3690 (69.8)	824 (66.5)	2156 (72.8)	710 (65.4)
Attitude towards mandatory COVID-19 vaccination for all people aged 12 and above, N (%)				
Negative attitude	2398 (45.4)	602 (48.5)	1258 (42.5)	538 (49.5)
Positive attitude	2889 (54.6)	638 (51.5)	1703 (57.5)	548 (50.5)
Attitude towards mandatory COVID-19 vaccination for admission to schools and universities, N (%)				
Negative attitude	2206 (41.7)	586 (47.3)	1125 (38.0)	495 (45.6)
Positive attitude	3081 (58.3)	654 (52.7)	1836 (62.0)	591 (54.4)

**Table 3:** Multivariable logistic regression models of positive student attitudes towards mandatory COVID-19 vaccination for healthcare workers (Model 1), for all people aged 12 or above (Model 2), and for admission to schools and universities (Model 3) (N = 5287).

	Model 1			Model 2			Model 3		
	aOR	(95% CI)	*p*-Value	aOR	(95% CI)	*p*-Value	aOR	(95% CI)	*p*-Value
Survey period									
September–October 2021	Ref.			Ref.			Ref.		
November–December 2021	1.08	(0.91–1.28)	0.371	1.09	(0.94–1.27)	0.247	**1.28**	**(1.10–1.49)**	**0.002**
January–February 2022	1.01	(0.82–1.23)	0.938	1.07	(0.89–1.29)	0.491	**1.21**	**(1.01–1.46)**	**0.045**
Vaccination status									
Unvaccinated	Ref.			Ref.			Ref.		
Vaccinated	**2.47**	**(1.36–4.48)**	**0.003**	1.14	(0.61–2.10)	0.684	1.81	(0.96–3.44)	0.068
Age	1.00	(0.98–1.01)	0.559	1.01	(0.99–1.03)	0.053	1.01	(0.99–1.03)	0.055
Gender									
Female	Ref.			Ref.			Ref.		
Male	0.96	(0.83–1.11)	0.603	1.04	(0.91–1.19)	0.585	1.06	(0.93–1.22)	0.369
Nationality									
Italian	Ref.			Ref.			Ref.		
Non-Italian	0.86	(0.70–1.06)	0.168	0.93	(0.76–1.13)	0.459	0.90	(0.74–1.10)	0.304
Area of study									
Healthcare	Ref.			Ref.			Ref.		
Science & Technology	0.88	(0.75–1.05)	0.157	0.86	(0.74–1.01)	0.061	**0.85**	**(0.73–0.99)**	**0.044**
Social Sciences & Humanities	0.87	(0.73–1.03)	0.104	**0.81**	**(0.69–0.94)**	**0.006**	**0.80**	**(0.69–0.93)**	**0.005**
Year of study									
First or second	Ref.			Ref.			Ref.		
Third or above	1.00	(0.86–1.15)	0.959	1.09	(0.96–1.25)	0.185	1.11	(0.97–1.27)	0.138
Finances									
Many or some difficulties	Ref.			Ref.			Ref.		
Managing well enough or very well	1.04	(0.90–1.20)	0.609	1.04	(0.91–1.18)	0.610	1.07	(0.93–1.22)	0.331
Previous COVID-19 infection									
No infection	Ref.			Ref.			Ref.		
Asymptomatic or mild symptoms	0.86	(0.69–1.09)	0.208	0.89	(0.72–1.10)	0.288	0.96	(0.77–1.19)	0.720
Moderate or severe symptoms	0.92	(0.54–1.59)	0.776	0.87	(0.52–1.46)	0.603	1.10	(0.65–1.84)	0.730
Perceived COVID-19 severity	**1.05**	**(1.01–1.10)**	**0.027**	**1.05**	**(1.01–1.10)**	**0.015**	**1.08**	**(1.04–1.13)**	**<0.001**
Concern about the COVID-19 emergency	1.04	(0.99–1.09)	0.064	**1.08**	**(1.04–1.13)**	**<0.001**	**1.09**	**(1.04–1.13)**	**<0.001**
Being afraid of becoming infected	1.01	(0.98–1.05)	0.581	1.03	(0.99–1.06)	0.057	**1.06**	**(1.03–1.10)**	**<0.001**
Being afraid of infecting people in the community	**1.10**	**(1.06–1.14)**	**<0.001**	**1.10**	**(1.06–1.14)**	**<0.001**	**1.08**	**(1.04–1.12)**	**<0.001**
Viewing the vaccine as an effective way to end the pandemic	**1.57**	**(1.49–1.65)**	**<0.001**	**1.56**	**(1.48–1.64)**	**<0.001**	**1.49**	**(1.42–1.57)**	**<0.001**

aOR: adjusted Odds Ratio. CI: Confidence Interval. COVID-19: Coronavirus Disease 2019. Significant predictors are in bold.

## Data Availability

The data presented in this study are available on request from the corresponding author.

## Supplementary Materials

The following Supporting Information can be downloaded at: https://www.mdpi.com/article/10.3390/vaccines11040721/s1, Table S1: Students’ sociodemographic characteristics by attitude towards mandatory COVID-19 vaccination; Table S2: Students’ COVID-19 experience and risk perceptions by attitude towards mandatory COVID-19 vaccination.
