# Supplementary material for: Attitudes of University Students towards Mandatory COVID-19 Vaccination Policies: A Cross-Sectional Survey in Rome, Italy
Source: Vaccines (Basel). 2023 Mar 23;11(4):721. doi: 10.3390/vaccines11040721 (PMC10141490; doi:10.3390/vaccines11040721)
Supplement: Supplementary file 1 [file vaccines-11-00721-s001.zip › vaccines-2286996-supplementary.pdf]

Supplementary Table S1. Students' sociodemographic characteristics by attitude towards mandatory COVID-19 vaccination (N = 5287). Results are expressed as mean (standard deviation, SD), median (interquartile range, IQR), or frequency (percentage, %).

	Attitude towards mandatory COVID-19 vaccination for healthcare workers			Attitude towards mandatory COVID-19 vaccination for all people aged 12 or above			Attitude towards mandatory COVID-19 vaccination as a requirement for admission to schools and universities		
	Negative attitude N=1597	Positive attitude N=3690	*p*-Value	Negative attitude N=2398	Positive attitude N=2889	*p*-Value	Negative attitude N=2206	Positive attitude N=3081	*p*-Value
Survey period, N (%)			<0.001			<0.001			<0.001
September-October 2021	416 (26.0)	824 (22.3)		602 (25.1)	638 (22.1)		586 (26.6)	654 (21.2)	
November-December 2021	805 (50.4)	2156 (58.4)		1258 (52.5)	1703 (58.9)		1125 (51.0)	1836 (59.6)	
January-February 2022	376 (23.5)	710 (19.2)		538 (22.4)	548 (19.0)		495 (22.4)	591 (19.2)	
Age, years									
Mean (SD)	24.3 (5.2)	23.8 (4.1)	<0.001	24.0 (4.8)	23.9 (4.2)	0.450	24.0 (4.7)	23.9 (4.3)	0.330
Median (IQR)	23.2 (21.3-25.6)	23.0 (21.2-25.0)	0.013	23.0 (21.1-25.3)	23.1 (21.3-25.1)	0.220	23.0 (21.2-25.4)	23.1 (21.3-25.1)	0.950
Gender, N (%)			<0.001			<0.001			<0.001
Female	1000 (62.6)	2580 (69.9)		1546 (64.5)	2034 (70.4)		1415 (64.1)	2165 (70.3)	
Male	597 (37.4)	1110 (30.1)		852 (35.5)	855 (29.6)		791 (35.9)	916 (29.7)	
Nationality, N (%)			<0.001			<0.001			<0.001
Italian	1304 (81.7)	3328 (90.2)		2021 (84.3)	2611 (90.4)		1845 (83.6)	2787 (90.5)	
Non-Italian	293 (18.3)	362 (9.8)		377 (15.7)	278 (9.6)		361 (16.4)	294 (9.5)	
Area of study, N (%)			<0.001			<0.001			<0.001
Healthcare	534 (33.4)	1460 (39.6)		816 (34.0)	1178 (40.8)		741 (33.6)	1253 (40.7)	
Science & Technology	513 (32.1)	1115 (30.2)		763 (31.8)	865 (29.9)		705 (32.0)	923 (30.0)	
Social Sciences & Humanities	550 (34.4)	1115 (30.2)		819 (34.2)	846 (29.3)		760 (34.5)	905 (29.4)	
Year of study, N (%)			0.270			0.005			0.005
First or second	1011 (63.3)	2277 (61.7)		1541 (64.3)	1747 (60.5)		1421 (64.4)	1867 (60.6)	
Third or above	586 (36.7)	1413 (38.3)		857 (35.7)	1142 (39.5)		785 (35.6)	1214 (39.4)	
Finances, N (%)			<0.001			0.065			0.062
Many difficuties	93 (5.8)	144 (3.9)		119 (5.0)	118 (4.1)		108 (4.9)	129 (4.2)	
Some difficulties	424 (26.5)	915 (24.8)		631 (26.3)	708 (24.5)		593 (26.9)	746 (24.2)	
Managing well enough	832 (52.1)	1921 (52.1)		1241 (51.8)	1512 (52.3)		1122 (50.9)	1631 (52.9)	

Managing very well	248 (15.5)	710 (19.2)		407 (17.0)	551 (19.1)		383 (17.4)	575 (18.7)	
Previous COVID-19 infection, N (%)			<0.001			<0.001			<0.001
No infection	1367 (85.6)	3342 (90.6)		2085 (86.9)	2624 (90.8)		1919 (87.0)	2790 (90.6)	
Asymptomatic or mild symptoms	200 (12.5)	301 (8.2)		270 (11.3)	231 (8.0)		250 (11.3)	251 (8.1)	
Moderate or severe symptoms	30 (1.9)	47 (1.3)		43 (1.8)	34 (1.2)		37 (1.7)	40 (1.3)	

COVID-19: coronavirus disease 2019.

Pearson’s chi-squared test for categorical variables and Mann-Whitney U test for continuous variables.

Supplementary Table S2. Students' COVID-19 experience and risk perceptions by attitude towards mandatory COVID-19 vaccination (N = 5287). Results are expressed as mean (standard deviation, SD) or frequency (percentage, %).

	Attitude towards mandatory COVID-19 vaccination for healthcare workers			Attitude towards mandatory COVID-19 vaccination for all people aged 12 or above			Attitude towards mandatory COVID-19 vaccination as a requirement for admission to schools and universities		
	Negative attitude N=1597	Positive attitude N=3690	*p*-Value	Negative attitude N=2398	Positive attitude N=2889	*p*-Value	Negative attitude N=2206	Positive attitude N=3081	*p*-Value
Vaccination status, N (%)			<0.001			<0.001			<0.001
Unvaccinated	84 (5.3)	19 (0.5)		85 (3.5)	18 (0.6)		88 (4.0)	15 (0.5)	
Vaccinated	1513 (94.7)	3671 (99.5)		2313 (96.5)	2871 (99.4)		2118 (96.0)	3066 (99.5)	
Reasons for not getting vaccinated (N = 103), N (%)			0.004			0.011			<0.001
I am suffering from a clinical condition with contraindications to COVID-19 vaccination/ waiting for further medical assessment	14 (16.7)	3 (15.8)		14 (16.5)	3 (16.7)		14 (15.9)	3 (20.0)	
I've already had COVID-19	16 (19.0)	5 (26.3)		17 (20.0)	4 (22.2)		17 (19.3)	4 (26.7)	
I've booked the vaccination/I am waiting to get vaccinated	6 (7.1)	6 (31.6)		6 (7.1)	6 (33.3)		6 (6.8)	6 (40.0)	
I don't consider myself at risk/prefer to obtain natural immunity to COVID-19	13 (15.5)	2 (10.5)		14 (16.5)	1 (5.6)		15 (17.0)	0 (0.0)	
I don't believe in the safety/effectiveness of vaccines against COVID-19	32 (38.1)	1 (5.3)		31 (36.5)	2 (11.1)		33 (37.5)	0 (0.0)	
No reason given	3 (3.6)	2 (10.5)		3 (3.5)	2 (11.1)		3 (3.4)	2 (13.3)	
Vaccine-adverse events (N= 5184), N (%)			0.090			0.330			0.250
No adverse events	550 (36.4)	1242 (33.8)		819 (35.4)	973 (33.9)		758 (35.8)	1034 (33.7)	
Mild adverse events	820 (54.2)	2022 (55.1)		1262 (54.6)	1580 (55.0)		1146 (54.1)	1696 (55.3)	

Moderate or severe adverse events	143 (9.5)	407 (11.1)		232 (10.0)	318 (11.1)		214 (10.1)	336 (11.0)	
Perceived COVID-19 severity, mean (SD)	6.9 (2.2)	7.9 (1.7)	<0.001	7.1 (2.0)	8.0 (1.7)	<0.001	7.0 (2.0)	8.0 (1.7)	<0.001
Concern about the COVID-19 emergency, mean (SD)	6.7 (2.3)	7.8 (1.8)	<0.001	6.9 (2.1)	7.9 (1.8)	<0.001	6.8 (2.1)	7.9 (1.8)	<0.001
Being afraid of infecting people in the community, mean (SD)	6.8 (2.6)	8.5 (2.0)	<0.001	7.2 (2.5)	8.7 (1.9)	<0.001	7.2 (2.5)	8.6 (2.0)	<0.001
Being afraid of becoming infected, mean (SD)	6.4 (2.7)	7.9 (2.3)	<0.001	6.7 (2.6)	8.1 (2.3)	<0.001	6.5 (2.6)	8.1 (2.3)	<0.001
Viewing the vaccine as an effective way to end the pandemic, mean (SD)	7.5 (2.5)	9.4 (1.3)	<0.001	8.0 (2.4)	9.5 (1.1)	<0.001	7.9 (2.4)	9.5 (1.2)	<0.001

COVID-19: coronavirus disease 2019.
Pearson's chi-squared test or Fisher's exact test for categorical variables and Mann-Whitney U test for continuous variables.
